# Supplementary material for: Collider bias undermines our understanding of COVID-19 disease risk and severity
Source: Nat Commun. 2020 Nov 12;11:5749. doi: 10.1038/s41467-020-19478-2 (PMC7665028; doi:10.1038/s41467-020-19478-2)
Supplement: Supplementary file 1 — Supplementary Information [file 41467_2020_19478_MOESM1_ESM.pdf]

Supplementary Information: UK Biobank example of collider bias in Covid-19 test data

Contents

About this document 1
Read in the data 2
Unweighted associations 3
Illustration of collider bias 4
Inverse probability weights in a nested sample 6
Comparing weighted and unweighted associations between risk factors and testing positive 14
Sensitivity analyses for non-nested samples 15
Summary 18
Supplementary references 19

About this document

This document forms part of the analysis used in the paper:

Collider bias undermines our understanding of COVID-19 disease risk and severity. Gareth Griffith, Tim T Morris, Matt Tudball, Annie Herbert, Giulia Mancano, Lindsey Pike, Gemma C Sharp, Jonathan Sterne, Tom M Palmer, George Davey Smith, Kate Tilling, Luisa Zuccolo, Neil M Davies, Gibran Hemani

It is hosted at https://github.com/MRCIEU/ukbb-covid-collider.

Here we show a set of analyses to illustrate collider bias induced by non-random testing of Covid-19 status amongst the UK Biobank participants, and some approaches to adjust for the bias. The methods are described in further detail in Griffith et al. (2020).

The following variables from the UK biobank phenotype data are used:

- `34-0.0` - Year of birth (converted into age for this analysis)
- `31-0.0` - Sex (male = 1, female = 0)
- `23104-0.0` - Body mass index (BMI)

Also, the linked Covid-19 freeze from `2020-06-05` is used to identify which individuals have been tested and tested positive.

In the analysis that follows, we will be estimating the association between testing positive for Covid-19 and the risk factors age, sex and BMI. The key concern with such an analysis is that we only observe test results among individuals who have received a test. SARS-CoV-2 infection and the risk factors themselves will influence the likelihood of receiving a test, which could induce spurious associations among them when we condition on receiving a test. We will explore inverse probability weighting and sensitivity analyses to address the potential collider bias.

Read in the data

```
suppressMessages(suppressPackageStartupMessages({
  library(knitr)
  library(dplyr)
  library(ggplot2)
  library(bootsens)
}))

knitr::opts_chunk$set(warning=FALSE, message=FALSE, echo=TRUE, cache=TRUE)
```

```
load("data/dat.rdata")
dat <- dat[complete.cases(dat[,c("age","sex","bmi","tested")]), ]
str(dat)
```

```
## tibble [492,392 x 5] (S3: tbl_df/tbl/data.frame)
##  $ age     : num [1:492392] 74 77 73 73 76 55 69 75 78 73 ...
##  $ sex     : int [1:492392] 0 1 1 0 1 1 0 0 0 0 ...
##  $ bmi     : num [1:492392] 20.8 27.5 28.6 27.3 26.5 26.5 30.9 24 23.2 31.8 ...
##  $ tested  : num [1:492392] 0 0 0 0 0 0 0 0 0 0 ...
##  $ positive: num [1:492392] NA NA NA NA NA NA NA NA NA NA ...
```

Summaries:

```
hist(dat$age)
```

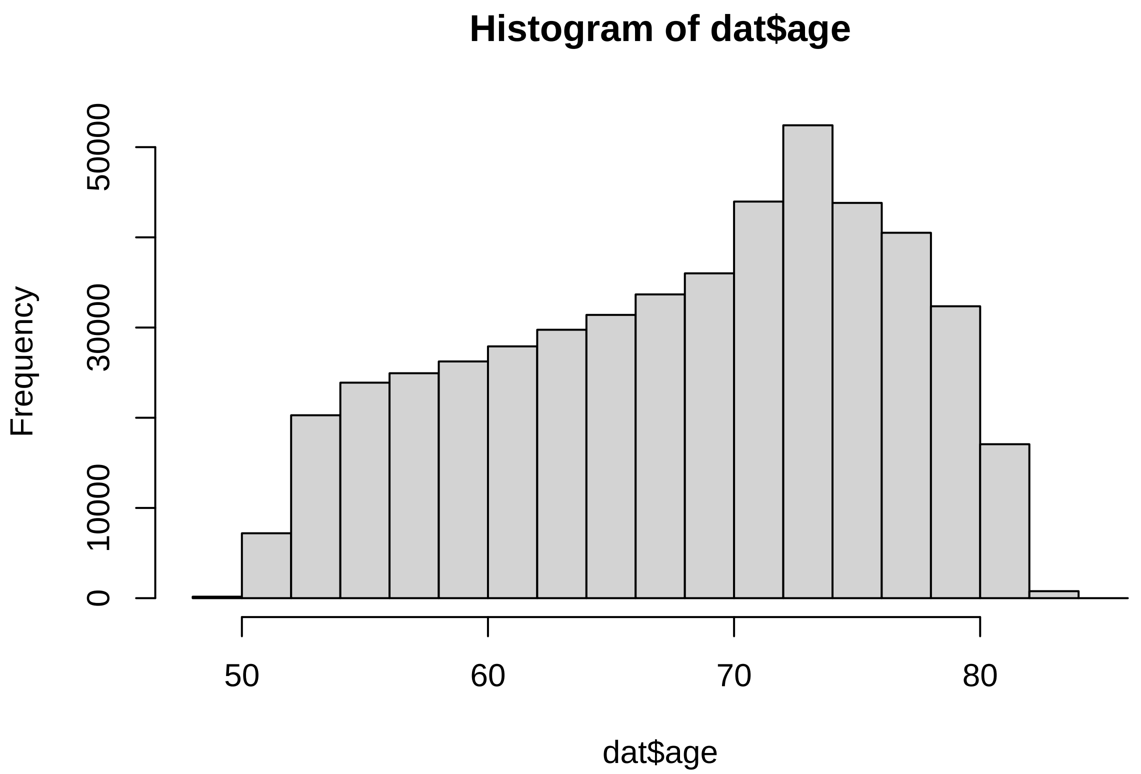


```
table(dat$sex) / nrow(dat)
```

```
##
##         0         1
## 0.5448383 0.4551617
```

How many individuals tested:

```
table(dat$tested)
```

```
##
##      0      1
## 486488   5904
```

How many individuals tested positive:

```
table(dat$positive)
```

```
##
##    0    1
## 4475 1429
```

Unweighted associations

The most basic approach is to report the raw pairwise associations between the risk factors and testing positive.

```
assoc <- data.frame(matrix(nrow=3, ncol=3))
names(assoc) <- c("risk_factor", "beta", "se")
assoc$risk_factor <- c("sex", "age", "bmi")

mod <- summary(lm(positive ~ sex, dat))
assoc[assoc$risk_factor=="sex",c("beta","se")] <-  coefficients(mod)["sex",c("Estimate","Std. Error")]

mod <- summary(lm(positive ~ age, dat))
assoc[assoc$risk_factor=="age",c("beta","se")] <-  coefficients(mod)["age",c("Estimate","Std. Error")]

mod <- summary(lm(positive ~ bmi, dat))
assoc[assoc$risk_factor=="bmi",c("beta","se")] <-  coefficients(mod)["bmi",c("Estimate","Std. Error")]

ggplot(assoc, aes(risk_factor, beta)) +
  geom_errorbar(
    aes(ymin = beta-1.96*se,
        ymax = beta+1.96*se),
    width = 0.1, size=1, color="#00BFC4") +
geom_point(size=3, shape=21, fill="#00BFC4") +
geom_hline(yintercept=0, linetype="dashed", color = "#575863") +
scale_x_discrete(labels=c("Age", "BMI", "Sex")) +
xlab("") + ylab("Value of association") +
coord_flip()
```

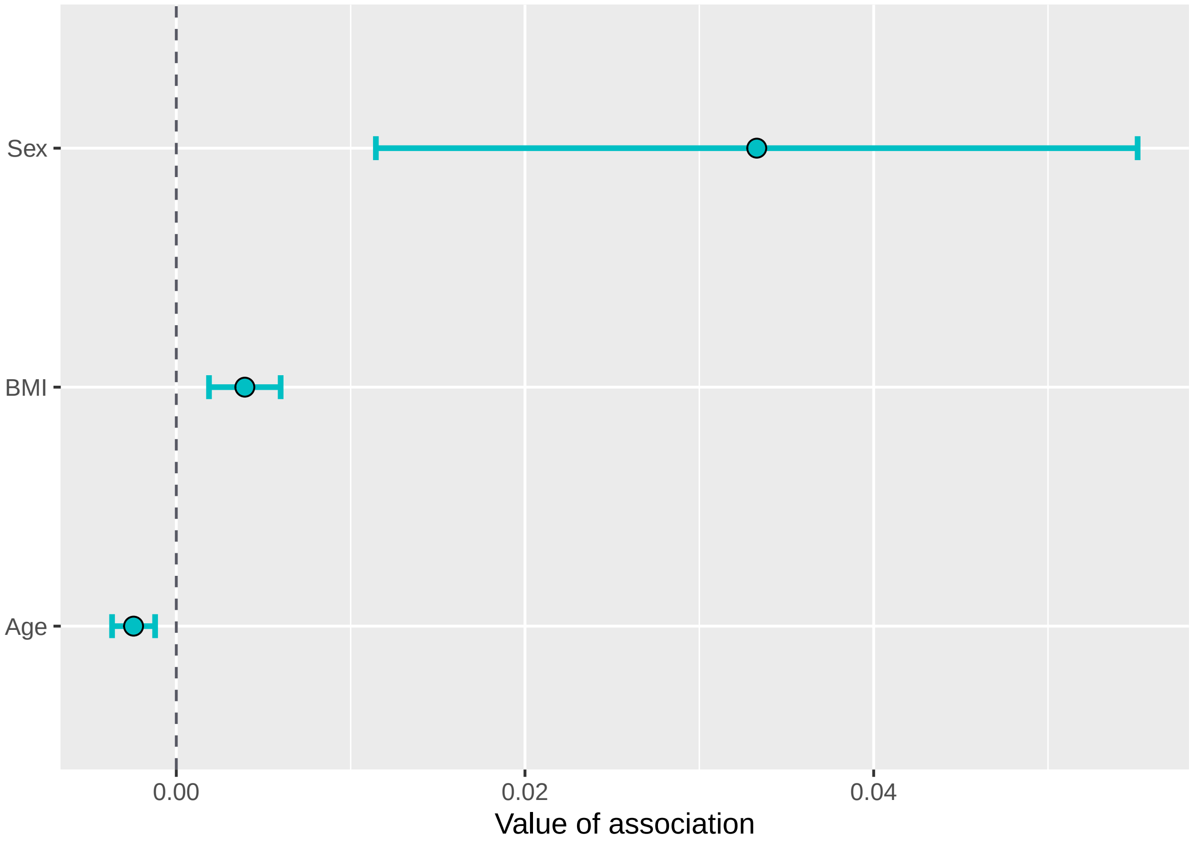

However, it is unclear to what extent these associations may be distorted by collider bias.

Illustration of collider bias

An simple exploratory exercise is to compare full sample and sub-sample associations among the variables that are observed in both, namely, age, sex and BMI.

We begin by exploring their relationship with receiving a test.

```
summary(glm(tested ~ age + sex + bmi, data=dat, family="binomial"))
```

```
##
## Call:
## glm(formula = tested ~ age + sex + bmi, family = "binomial",
##     data = dat)
##
## Deviance Residuals:
##     Min       1Q   Median       3Q      Max
## -0.3405  -0.1628  -0.1527  -0.1434   3.1402
##
## Coefficients:
##              Estimate Std. Error z value Pr(>|z|)
## (Intercept) -5.922371   0.132640 -44.650  < 2e-16 ***
## age          0.006487   0.001634   3.969 7.22e-05 ***
## sex          0.103816   0.026224   3.959 7.53e-05 ***
## bmi          0.036462   0.002500  14.585  < 2e-16 ***
## ---
```

```
## Signif. codes:  0 '***' 0.001 '**' 0.01 '*' 0.05 '.' 0.1 ' ' 1
##
## (Dispersion parameter for binomial family taken to be 1)
##
##     Null deviance: 63971  on 492391  degrees of freedom
## Residual deviance: 63730  on 492388  degrees of freedom
## AIC: 63738
##
## Number of Fisher Scoring iterations: 7
```

Therefore, we would expect the relationships between these variables to be skewed within the tested sample. For example, this is the relationship between age and sex in the full sample:

```
mod1 <- summary(lm(age ~ sex, dat))
coefficients(mod1) %>% kable
```

	Estimate	Std. Error	t value	Pr(>|t|)
(Intercept)	68.2695118	0.0156525	4361.57945	0
sex	0.3703387	0.0232006	15.96243	0

And here it is among those who received a test:

```
mod2 <- summary(lm(age ~ sex, dat, subset=dat$tested==1))
coefficients(mod2) %>% kable
```

	Estimate	Std. Error	t value	Pr(>|t|)
(Intercept)	67.67524	0.1589147	425.85894	0
sex	2.54350	0.2278876	11.16121	0

Note that the association is around **7 times larger** in the tested subset.

Similarly, this is the overall association between age and BMI:

```
mod3 <- summary(lm(bmi ~ sex, dat))
coefficients(mod3) %>% kable
```

	Estimate	Std. Error	t value	Pr(>|t|)
(Intercept)	27.0942063	0.0092111	2941.48475	0
sex	0.7419068	0.0136530	54.34035	0

and here in the subsample:

```
mod4 <- summary(lm(bmi ~ sex, dat, subset=dat$tested==1))
coefficients(mod4) %>% kable
```

	Estimate	Std. Error	t value	Pr(>|t|)
(Intercept)	28.1546983	0.0963758	292.134651	0.0000000
sex	0.4174368	0.1382052	3.020413	0.0025351

where the association almost halves.

Inverse probability weights in a nested sample

The first approach we consider is inverse probability weighting. Since tested individuals are a subset of the entire sample, it is possible to generate probabilities of being included in the tested sub-sample based on the measured risk factors. The inverse of these probabilities can be used to weight the association estimate towards being representative of the full sample.

Note that, since testing positive is only measured within the tested sub-sample, we cannot include it in the weighting model. Therefore, for the inverse weighted estimates to be unbiased for the full-sample risk factor associations, we would need each individual's likelihood of being tested to be independent of whether they are infected with Covid-19 given their age, sex and BMI. Since this is unlikely to be true, we will explore sensitivity analyses later in the tutorial.

First, find which variables associate with being tested. Begin with marginal effects

```
mod5 <- glm(tested ~ age + sex + bmi, data=dat, family="binomial")
prs1 <- fitted.values(mod5)
coefficients(summary(mod5)) %>% kable
```

	Estimate	Std. Error	z value	Pr(>|z|)
(Intercept)	-5.9223706	0.1326401	-44.649915	0.00e+00
age	0.0064868	0.0016344	3.968823	7.22e-05
sex	0.1038164	0.0262241	3.958822	7.53e-05
bmi	0.0364623	0.0025000	14.585195	0.00e+00

It's important to also test for interactions between variables (Groenwold, Palmer, and Tilling 2020).

```
mod6 <- glm(tested ~ bmi + age + sex + bmi * age + bmi * sex + age * sex + age * bmi * sex, data=dat, fa
coefficients(summary(mod6)) %>% kable
```

	Estimate	Std. Error	z value	Pr(>|z|)
(Intercept)	-2.4386662	0.7600262	-3.208661	0.0013335
bmi	-0.0469310	0.0267885	-1.751907	0.0797899
age	-0.0452259	0.0112794	-4.009590	0.0000608
sex	-3.9881129	1.3111898	-3.041598	0.0023533
bmi:age	0.0012435	0.0003960	3.140162	0.0016885
bmi:sex	0.0604504	0.0458599	1.318153	0.1874523
age:sex	0.0598076	0.0189730	3.152243	0.0016202
bmi:age:sex	-0.0008918	0.0006627	-1.345629	0.1784222

Curiously, the marginal BMI effect appears to be captured by interaction terms only. Centering the marginal variables may be more appropriate when estimating interactions, as otherwise the interaction term is simply a multiplicative effect which is correlated to the marginal effects.

```
dat$bmixage <- scale(dat$bmi) * scale(dat$age)
dat$bmixsex <- scale(dat$bmi) * scale(dat$sex)
dat$agexsex <- scale(dat$age) * scale(dat$sex)
dat$agexsexxbmi <- scale(dat$age) * scale(dat$sex) * scale(dat$bmi)
mod7 <- glm(tested ~ bmi + age + sex + bmixage + bmixsex + agexsex + agexsexxbmi, data=dat, family="bin
coefficients(summary(mod7)) %>% kable
```

	Estimate	Std. Error	z value	Pr(>|z|)
(Intercept)	-5.8669485	0.1324589	-44.2926117	0.0000000

	Estimate	Std. Error	z value	Pr(>|z|)
bmi	0.0379081	0.0025664	14.7706579	0.0000000
age	0.0049735	0.0016671	2.9832590	0.0028520
sex	0.0890447	0.0271695	3.2773819	0.0010477
bmixage	0.0325030	0.0125774	2.5842439	0.0097593
bmixsex	-0.0013862	0.0124699	-0.1111627	0.9114873
agexsex	0.1427313	0.0134988	10.5736388	0.0000000
agexsexxbmi	-0.0172329	0.0128066	-1.3456290	0.1784222

Here the marginal effects are all retained.

We can generate the probabilities of each individual being tested based on these variables using:

```
prs2 <- fitted.values(mod7)
hist(prs2, breaks=100)
```

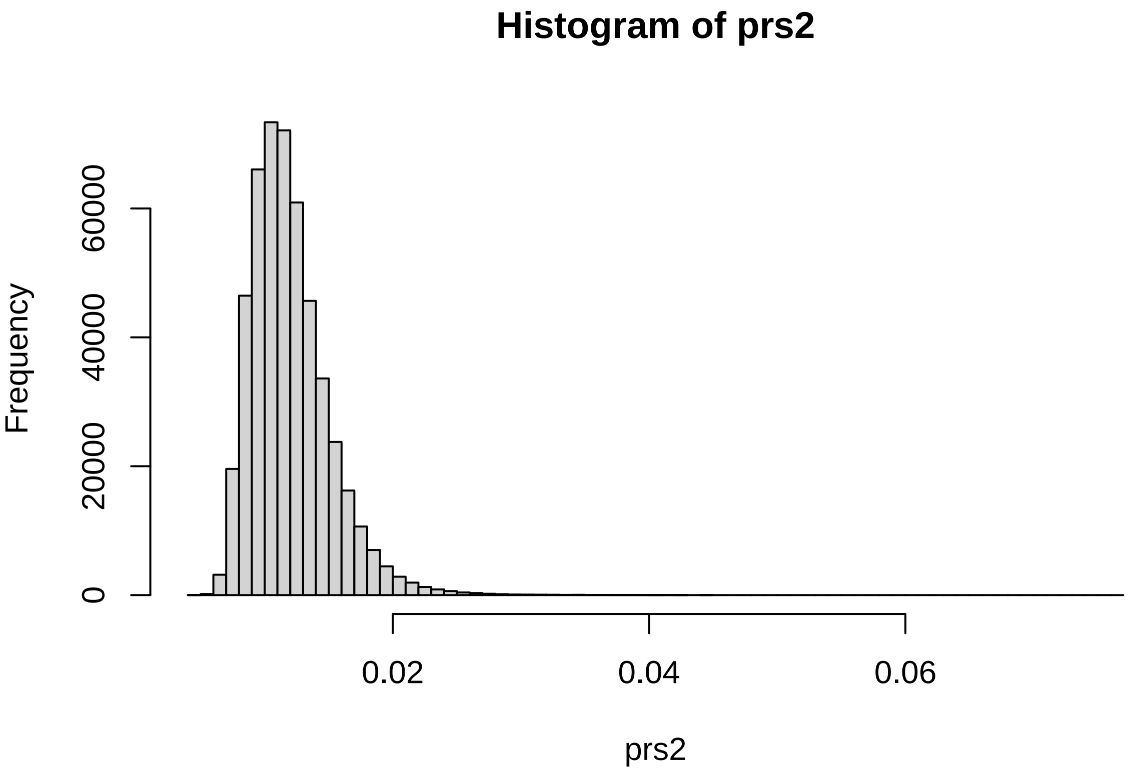

It is also important to consider non-linearities in the relationship between testing and age and BMI, which are continuous. There appears to be a quadratic relationship between age and testing and a cubic relationship between BMI and testing.

```
# Age plot
ggplot(dat[dat$age<82,],aes(x=age, y=tested)) +
  geom_smooth(method="gam",formula=y~s(x)) +
  xlab("Age") + ylab("Likelihood of Testing") +
  scale_x_continuous(breaks=seq(min(dat$age),82,by=2))
```

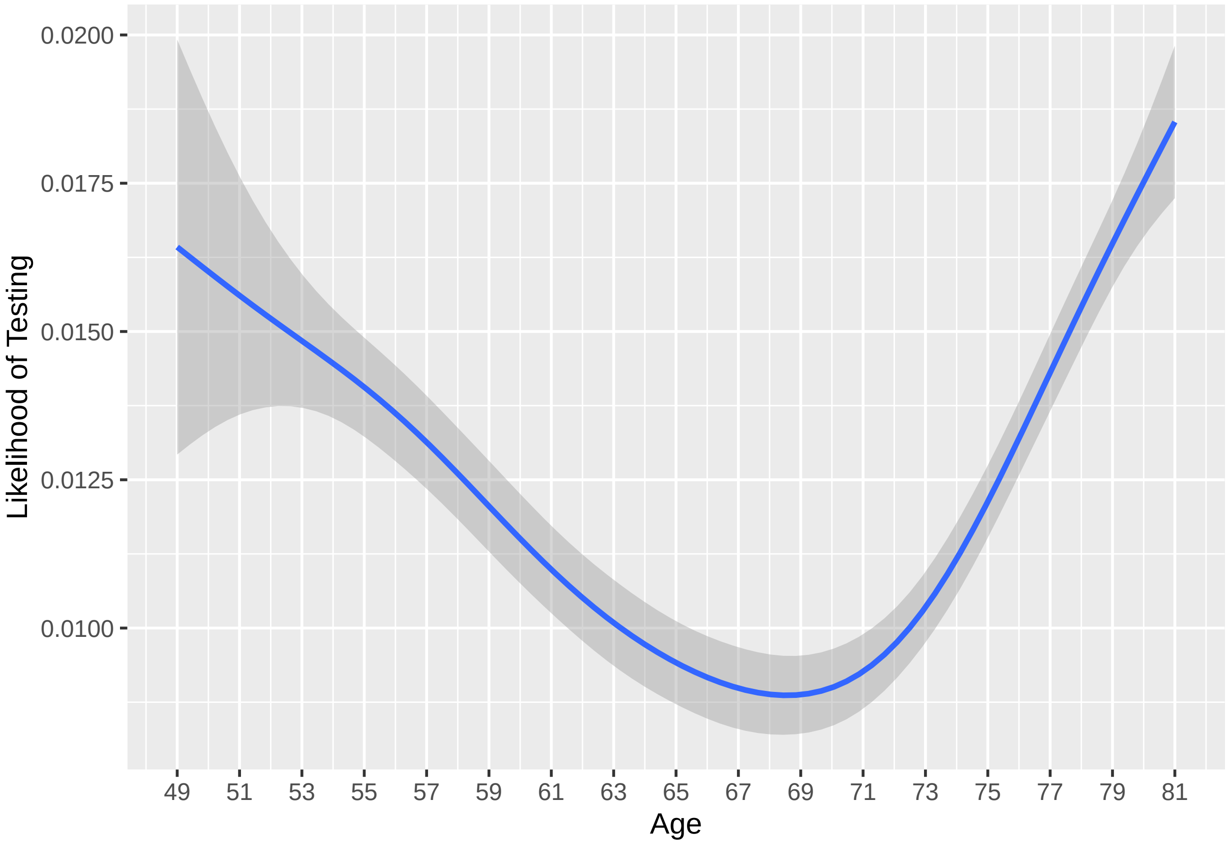


```
# BMI plot
ggplot(dat[dat$bmi<60,],aes(x=bmi, y=tested)) +
   geom_smooth(method="gam",formula=y~s(x)) +
  xlab("BMI") + ylab("Likelihood of Testing") +
  scale_x_continuous(breaks=seq(12,60,by=5))
```

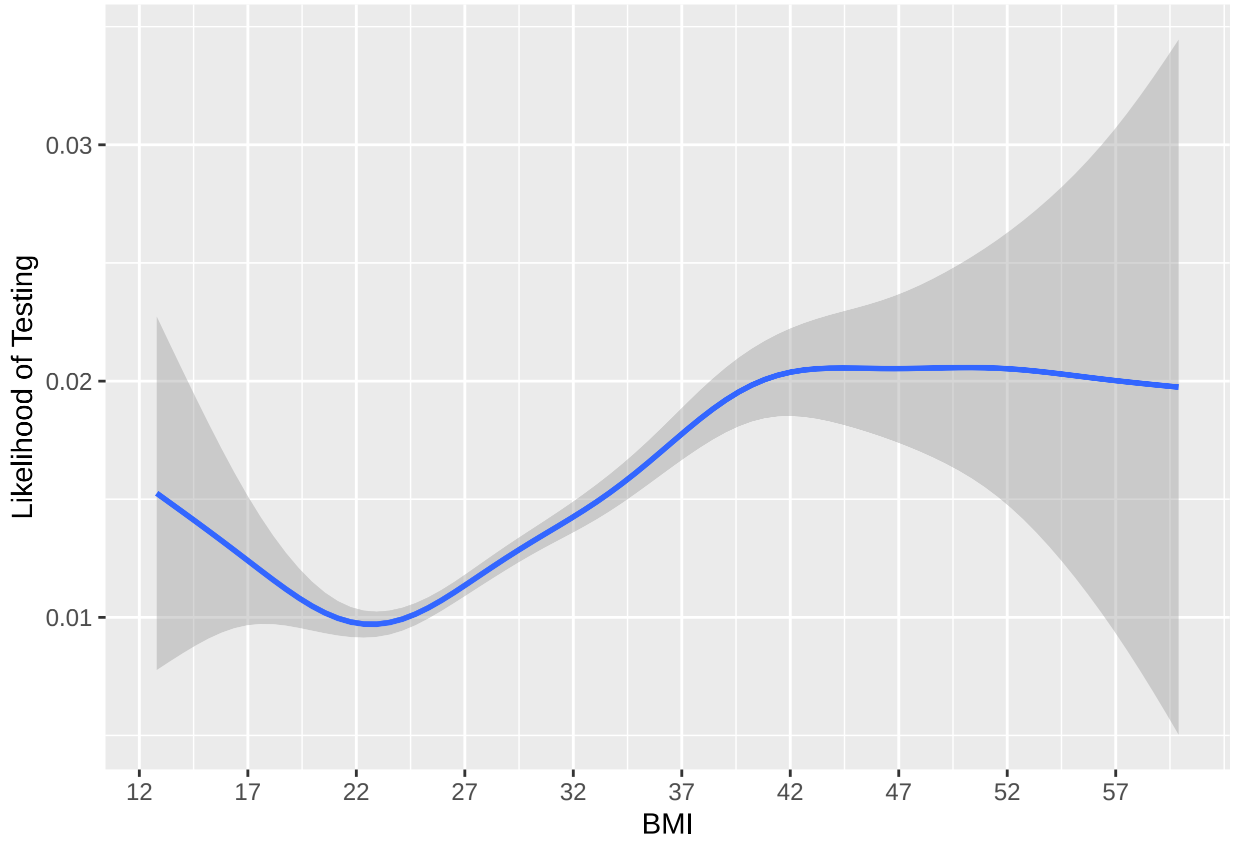

We select a logistic regression which is quadratic in age, cubic in BMI and contains all linear interactions between age, sex and BMI.

```
mod8 <- glm(tested ~ sex + poly(age, 2) + poly(bmi, 3) + bmixage + bmixsex + agexsex + agexsexxbmi, data
summary(mod8)
```

```
##
## Call:
## glm(formula = tested ~ sex + poly(age, 2) + poly(bmi, 3) + bmixage +
##     bmixsex + agexsex + agexsexxbmi, family = "binomial", data = dat)
##
## Deviance Residuals:
##     Min       1Q   Median       3Q      Max
## -0.3560  -0.1657  -0.1467  -0.1340   3.1318
##
## Coefficients:
##                  Estimate Std. Error  z value Pr(>|z|)
## (Intercept)     -4.505016   0.018849 -239.006  < 2e-16 ***
## sex              0.090564   0.027568    3.285  0.00102 **
## poly(age, 2)1   27.063717   8.678689    3.118  0.00182 **
## poly(age, 2)2  140.043970   8.604877   16.275  < 2e-16 ***
## poly(bmi, 3)1  125.971024   8.872164   14.198  < 2e-16 ***
## poly(bmi, 3)2   14.102663   8.422602    1.674  0.09406 .
## poly(bmi, 3)3  -28.860842   9.025124   -3.198  0.00138 **
## bmixage          0.019016   0.011397    1.669  0.09521 .
## bmixsex         -0.002089   0.012433   -0.168  0.86659
## agexsex          0.115827   0.012389    9.349  < 2e-16 ***
## agexsexxbmi     -0.011824   0.011538   -1.025  0.30547
```

```
## ---
## Signif. codes:  0 '***' 0.001 '**' 0.01 '*' 0.05 '.' 0.1 ' ' 1
##
## (Dispersion parameter for binomial family taken to be 1)
##
##     Null deviance: 63971  on 492391  degrees of freedom
## Residual deviance: 63342  on 492381  degrees of freedom
## AIC: 63364
##
## Number of Fisher Scoring iterations: 7
```

```
prs3 <- fitted.values(mod8)
hist(prs3, breaks=100)
```

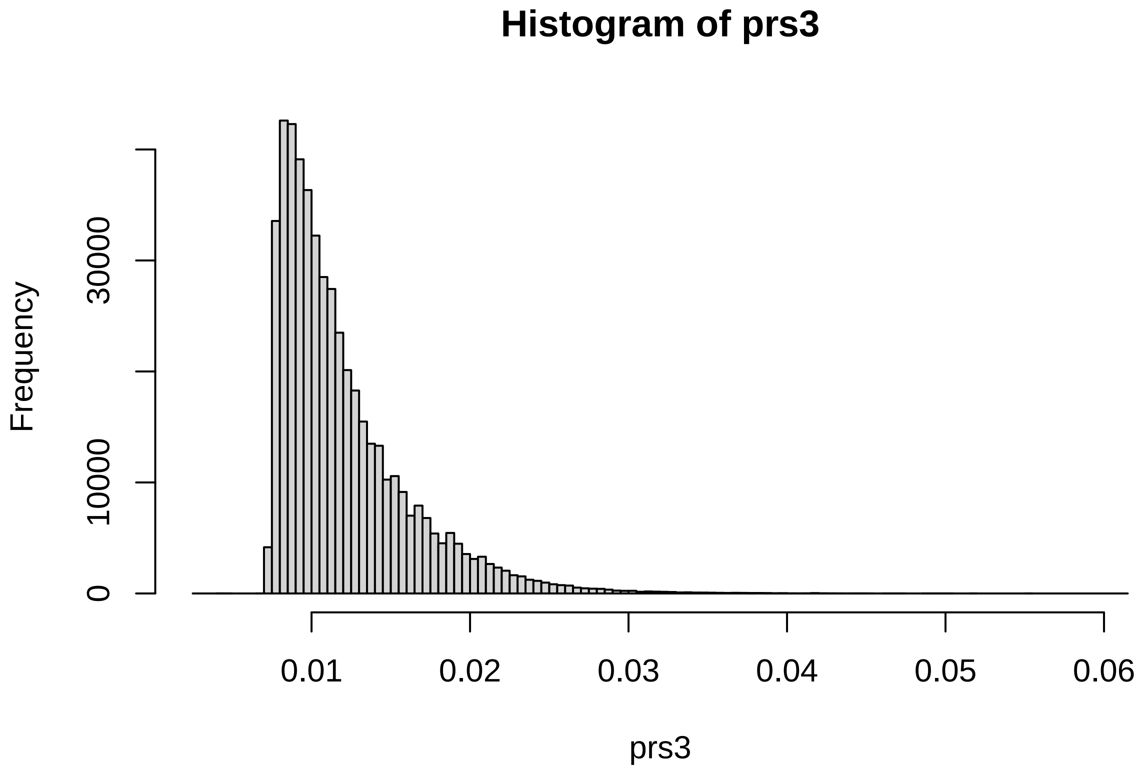

The probabilities are largely clustered close to zero, so when using the inverse of these probabilities for weights there can be potential instability that comes from dividing by small numbers. Stabilising the weight, by multiplying by the probability of being tested, can avoid this issue (Sayon-Orea et al. 2020).

```
p <- mean(as.numeric(dat$tested))
ipw1 <- ifelse(dat$tested==1, p/prs1, (1-p)/(1-prs1))
ipw2 <- ifelse(dat$tested==1, p/prs2, (1-p)/(1-prs2))
ipw3 <- ifelse(dat$tested==1, p/prs3, (1-p)/(1-prs3))
```

Validation exercise: Comparing full sample, unweighted and weighted associations among fully measured variables

Below we perform a validation exercise where we compare the full sample associations with the weighted associations for age, sex and BMI.

```
# Prepare output file
group <- expand.grid(c("as","ab","bs"),c(1,2,3,4,5))
out <- data.frame(cbind(group, matrix(nrow=15, ncol=2)))
names(out) <- c("assoc","model","beta","se")

# Age and sex association
# Full sample
mod <- summary(lm(age ~ sex, dat))
out[out$assoc=="as"&out$model==5,c("beta","se")] <- coefficients(mod)["sex",c("Estimate","Std. Error")]

# Tested subsample
mod <- summary(lm(age ~ sex, dat, subset=dat$tested==1))
out[out$assoc=="as"&out$model==4,c("beta","se")] <- coefficients(mod)["sex",c("Estimate","Std. Error")]

# Inverse weighted (linear, no interactions)
mod <- summary(lm(age ~ sex, dat, weight=ipw1, subset=tested==1))
out[out$assoc=="as"&out$model==1,c("beta","se")] <- coefficients(mod)["sex",c("Estimate","Std. Error")]

# Inverse weighted (linear, interactions)
mod <- summary(lm(age ~ sex, dat, weight=ipw2, subset=tested==1))
out[out$assoc=="as"&out$model==2,c("beta","se")] <- coefficients(mod)["sex",c("Estimate","Std. Error")]

# Inverse weighted (nonlinear, interactions)
mod <- summary(lm(age ~ sex, dat, weight=ipw3, subset=tested==1))
out[out$assoc=="as"&out$model==3,c("beta","se")] <- coefficients(mod)["sex",c("Estimate","Std. Error")]

# BMI and sex association
# Full sample
mod <- summary(lm(bmi ~ sex, dat))
out[out$assoc=="bs"&out$model==5,c("beta","se")] <- coefficients(mod)["sex",c("Estimate","Std. Error")]

# Tested subsample
mod <- summary(lm(bmi ~ sex, dat, subset=dat$tested==1))
out[out$assoc=="bs"&out$model==4,c("beta","se")] <- coefficients(mod)["sex",c("Estimate","Std. Error")]

# Inverse weighted (linear, no interactions)
mod <- summary(lm(bmi ~ sex, dat, weight=ipw1, subset=tested==1))
out[out$assoc=="bs"&out$model==1,c("beta","se")] <- coefficients(mod)["sex",c("Estimate","Std. Error")]

# Inverse weighted (linear, interactions)
mod <- summary(lm(bmi ~ sex, dat, weight=ipw2, subset=tested==1))
out[out$assoc=="bs"&out$model==2,c("beta","se")] <- coefficients(mod)["sex",c("Estimate","Std. Error")]

# Inverse weighted (nonlinear, interactions)
mod <- summary(lm(bmi ~ sex, dat, weight=ipw3, subset=tested==1))
out[out$assoc=="bs"&out$model==3,c("beta","se")] <- coefficients(mod)["sex",c("Estimate","Std. Error")]

# BMI and age association
# Full sample
mod <- summary(lm(bmi ~ age, dat))
out[out$assoc=="ab"&out$model==5,c("beta","se")] <- coefficients(mod)["age",c("Estimate","Std. Error")]

# Tested subsample
```

```
mod <- summary(lm(bmi ~ age, dat, subset=dat$tested==1))
out[out$assoc=="ab"&out$model==4,c("beta","se")] <- coefficients(mod)["age",c("Estimate","Std. Error")]

# Inverse weighted (linear, no interactions)
mod <- summary(lm(bmi ~ age, dat, weight=ipw1, subset=tested==1))
out[out$assoc=="ab"&out$model==1,c("beta","se")] <- coefficients(mod)["age",c("Estimate","Std. Error")]

# Inverse weighted (linear, interactions)
mod <- summary(lm(bmi ~ age, dat, weight=ipw2, subset=tested==1))
out[out$assoc=="ab"&out$model==2,c("beta","se")] <- coefficients(mod)["age",c("Estimate","Std. Error")]

# Inverse weighted (nonlinear, interactions)
mod <- summary(lm(bmi ~ age, dat, weight=ipw3, subset=tested==1))
out[out$assoc=="ab"&out$model==3,c("beta","se")] <- coefficients(mod)["age",c("Estimate","Std. Error")]
```

```
ggplot() + geom_errorbar(data=out[out$assoc=="as",], mapping=aes(x=as.factor(model), ymin=beta-1.96*se,
geom_point(data=out[out$assoc=="as",], mapping=aes(x=as.factor(model), y=beta), size=3, shape=21, fill=
```

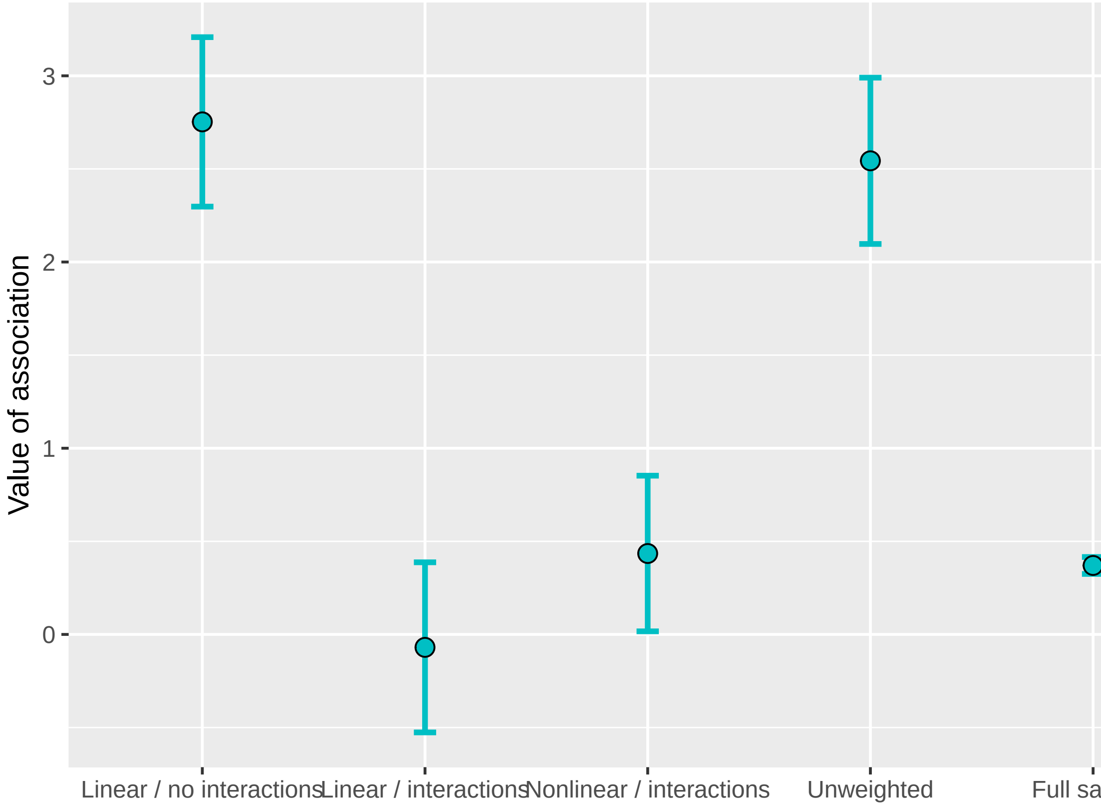

Age and sex association

```
ggplot() + geom_errorbar(data=out[out$assoc=="bs",], mapping=aes(x=as.factor(model), ymin=beta-1.96*se,
geom_point(data=out[out$assoc=="bs",], mapping=aes(x=as.factor(model), y=beta), size=3, shape=21, fill=
```

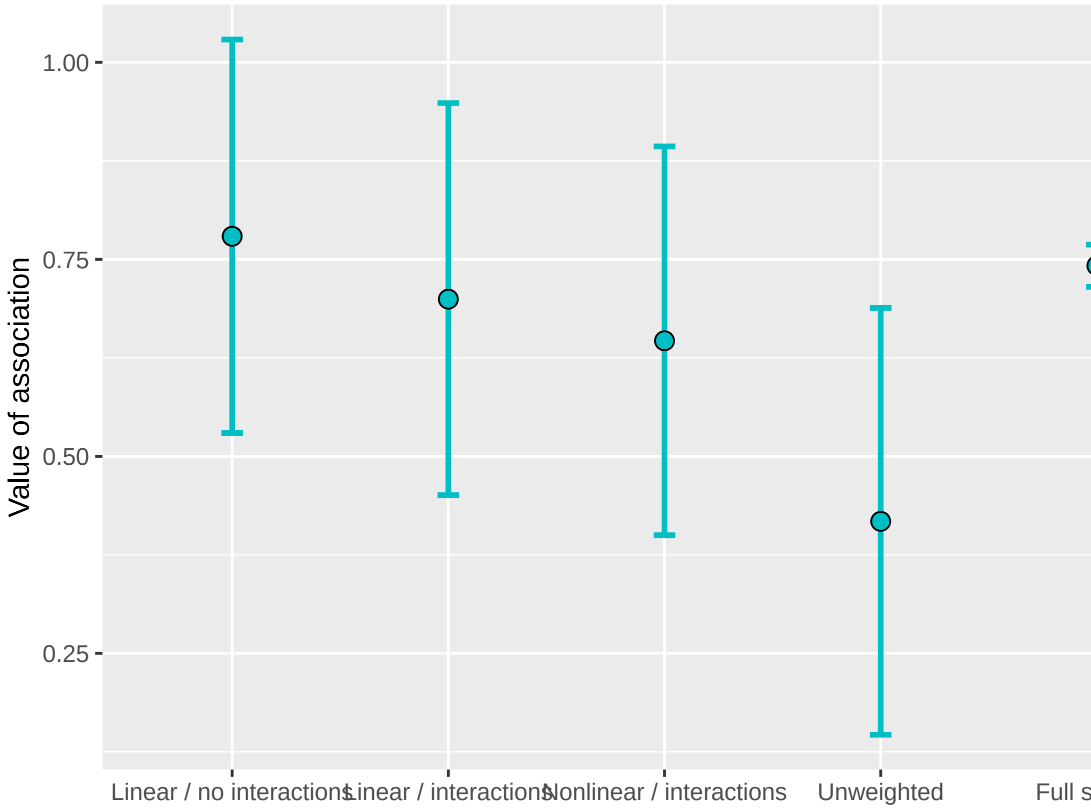

BMI and sex association

```
ggplot() + geom_errorbar(data=out[out$assoc=="ab",], mapping=aes(x=as.factor(model), ymin=beta-1.96*se,
geom_point(data=out[out$assoc=="ab",], mapping=aes(x=as.factor(model), y=beta), size=3, shape=21, fill=
```

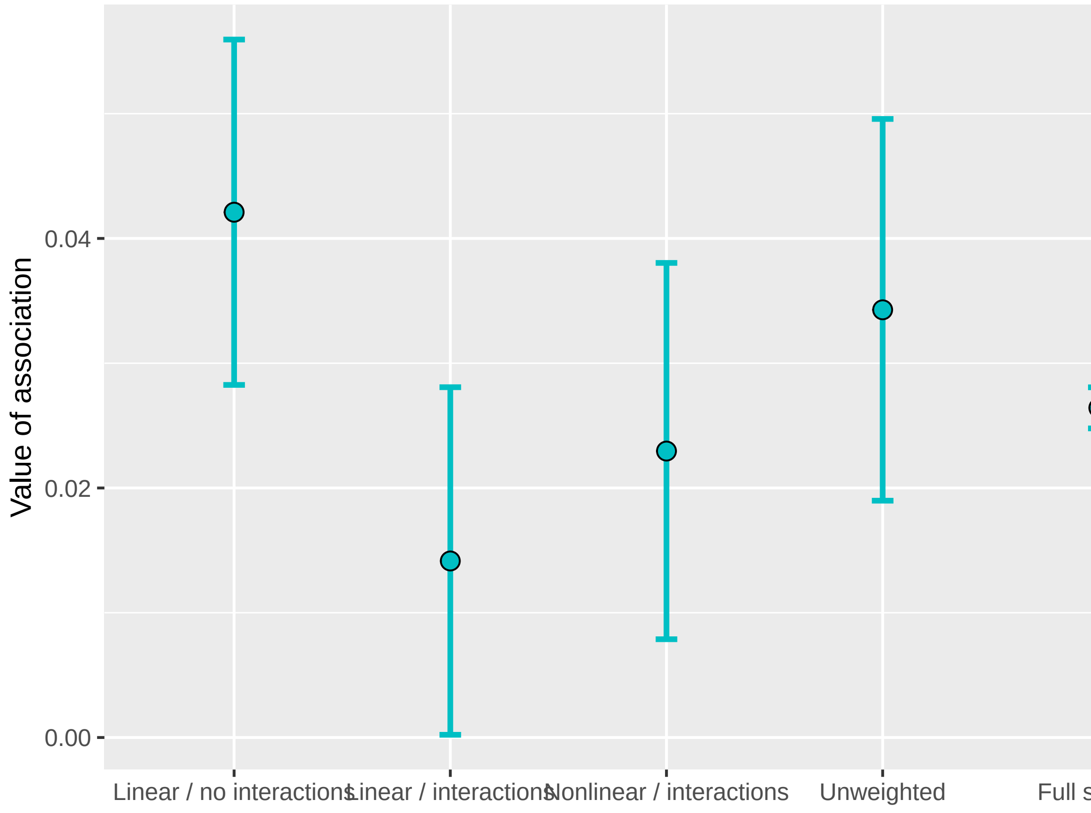

BMI and age association

Comparing weighted and unweighted associations between risk factors and testing positive

The previous validation exercise suggests that the full weighting model with interactions and non-linearities performs best at recovering the full sample associations among sex, age and BMI. While this does not mean it will necessarily recover the full sample associations between risk factors and testing positive, it is a useful starting point. Below we compare the weighted risk factor associations with the unweighted associations estimated earlier.

```
assoc2 <- data.frame(matrix(nrow=3, ncol=3))
names(assoc2) <- c("risk_factor", "beta", "se")
assoc2$risk_factor <- c("sex", "age", "bmi")

mod <- summary(lm(positive ~ sex, dat, weight=ipw3))
assoc2[assoc2$risk_factor=="sex",c("beta","se")] <-  coefficients(mod)["sex",c("Estimate","Std. Error")]

mod <- summary(lm(positive ~ age, dat, weight=ipw3))
assoc2[assoc2$risk_factor=="age",c("beta","se")] <-  coefficients(mod)["age",c("Estimate","Std. Error")]

mod <- summary(lm(positive ~ bmi, dat, weight=ipw3))
assoc2[assoc2$risk_factor=="bmi",c("beta","se")] <-  coefficients(mod)["bmi",c("Estimate","Std. Error")]

assoc <- rbind(assoc, assoc2)
assoc$model <- c(rep(1,3),rep(2,3))

ggplot(assoc, aes(risk_factor, beta)) +
  geom_errorbar(
```

```
    aes(ymin = beta-1.96*se,
        ymax = beta+1.96*se,
        color = as.factor(model)),
    position = position_dodge(0.3), size=1, width=0.1
  ) +
  xlab("") + ylab("Value of association") +
  geom_point(aes(color = as.factor(model)), size=3, position = position_dodge(0.3)) +
  geom_hline(yintercept=0, linetype="dashed", color = "#575863") +
  labs(colour="") +
  scale_color_manual(name="Estimates", labels=c("Unweighted", "Weighted"), values=c("#F8766D","#00BFC4"
  coord_flip()
```

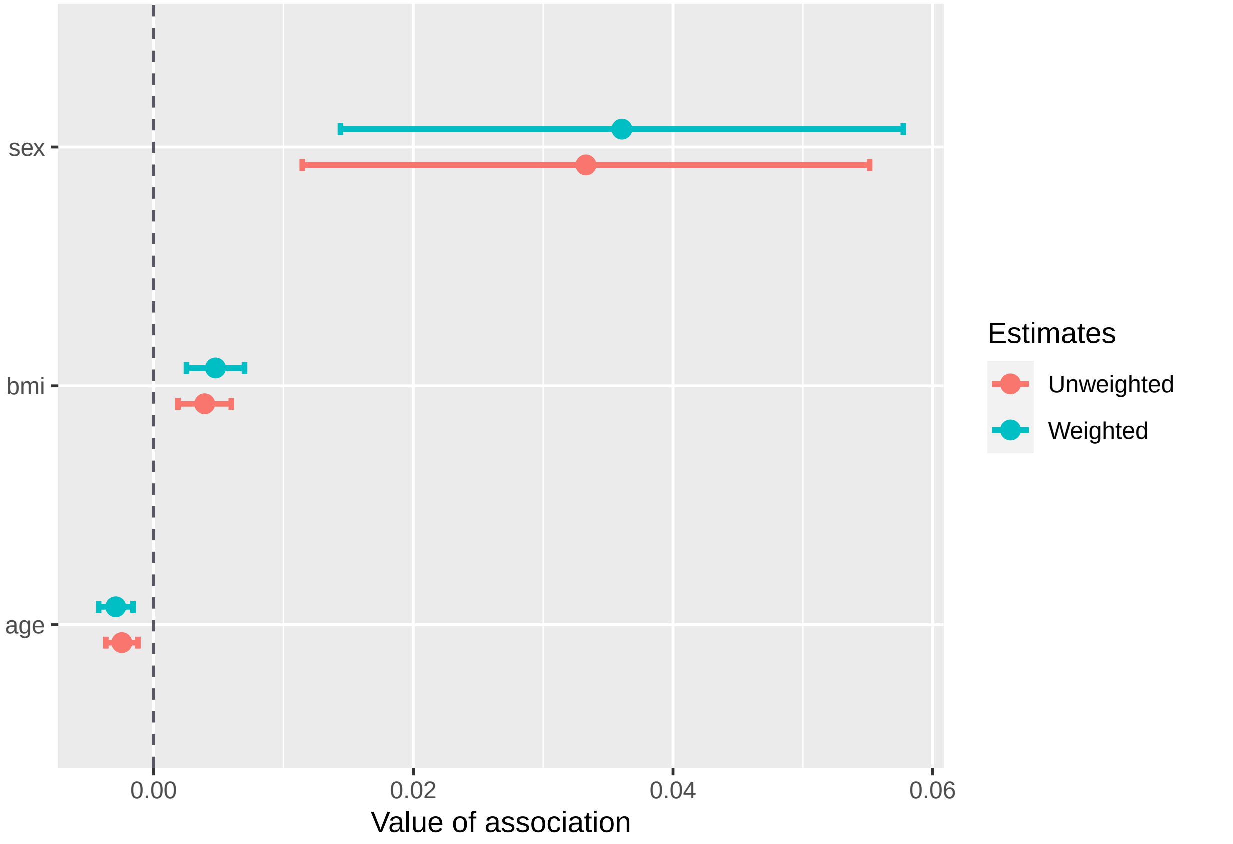

The weighted and unweighted associations are very similar. This could be indicative of no collider bias, however, it is more likely in this instance that the weights are misspecified due to the absence of the positive test variable. Below we explore sensitivity analyses for this misspecification.

Sensitivity analyses for non-nested samples

We now present two sensitivity analyses which can be used when direct estimation of the probability weights is limited or not possible. Zhao et al (2019) is most appropriately used when probability weights can be estimated but are likely to be misspecified (e.g. missing an important predictor). Tudball et al (2020) is most appropriately used when weights cannot be estimated but there is external information on the target population (e.g. survey response rate, population means).

We will attempt to estimate the association between sex and testing positive in the full UK Biobank population using data on the tested sub-sample.

Zhao, Small, and Bhattacharya (2019)

The sensitivity parameter in Zhao, Small, and Bhattacharya (2019) is the largest amount that the estimated weights differ from the true weights on the odds ratio scale. Given this parameter, this method provides an interval of possible values for means and associations with binary exposures.

In our example, misspecification is due to not including the positive test variable in the weight model. We choose as our sensitivity parameter an odds ratio of 1.1.

```
Gamma <- 1.1

dat$age2 <- dat$age^2; dat$bmi2 <- dat$bmi^2; dat$bmi3 <- dat$bmi^3

dat1 <- dat[dat$sex==1,]
sens1 <- bootsens::extrema.md(A=dat1$tested, Y=dat1$positive, X <- as.matrix(dat1[,!(names(dat1) %in% c

dat0 <- dat[dat$sex==0,]
sens0 <- bootsens::extrema.md(A=dat0$tested, Y=dat0$positive, X <- as.matrix(dat0[,!(names(dat0) %in% c

sens1_ci <- bootsens::bootsens.md(A=dat1$tested, Y=dat1$positive, X <- as.matrix(dat1[,!(names(dat1) %i

sens0_ci <- bootsens::bootsens.md(A=dat0$tested, Y=dat0$positive, X <- as.matrix(dat0[,!(names(dat0) %i

out1 <- list(
  beta_min=sens1[1]-sens0[2],
  beta_max=sens1[2]-sens0[1],
  beta_lower=sens1_ci[1]-sens0_ci[2],
  beta_upper=sens1_ci[2]-sens0_ci[1]
)

tibble(
    method="Zhao et al (2019)",
    lower_ci=round(out1$beta_lower,2),
    lower_bound=round(out1$beta_min,2),
    upper_bound=round(out1$beta_max,2),
    upper_ci=round(out1$beta_upper,2)
) %>% kable
```

method	lower_ci	lower_bound	upper_bound	upper_ci
Zhao et al (2019)	-0.06	-0.03	0.11	0.14

The resulting bounds indicate that even small amounts of misspecification can lead to considerable uncertainty. The bounds themselves indicate that collider bias from positive testing could either upward bias or downward bias the association between sex and testing positive. One reason these bounds are so wide is because this method is non-parametric, that is, it does not restrict the way in the true weights may differ from the estimated weights.

Tudball et al. (2020)

Suppose we only observe the tested sub-sample. Estimating probability weights is no longer possible since we do not observe anyone who has not been tested. The two sensitivity parameters in Tudball et al. (2020) are the smallest and largest probabilities of being tested (e.g. 1% and 90%). We then select a model for the

probability weights as before and this method places bounds on the possible association between sex and testing positive in the full UK Biobank population.

An advantage of this method is that we can place additional constraints on the bounds. Suppose we know the unconditional likelihood of being tested and the average age and BMI of UK Biobank participants. We can include these as constraints to ensure that the resulting bounds are consistent with these population values. In reality, we may have more or fewer constraints than this.

A non-parametric version of this method also exists but here we present the parametric version using a logistic model for the weights. The parametric version results in tighter bounds but carries the risk of misspecification of the weights.

```
mean_response1 <- mean(dat$tested[dat$sex==1])
mean_age1 <- mean(dat$age[dat$sex==1])
mean_bmi1 <- mean(dat$bmi[dat$sex==1])
mean_response0 <- mean(dat$tested[dat$sex==0])
mean_age0 <- mean(dat$age[dat$sex==0])
mean_bmi0 <- mean(dat$bmi[dat$sex==0])

dattest <- dat[complete.cases(dat), ]

D <- as.data.frame(dattest[,c('age','bmi','positive')])
D$agexbmi <- D$age*D$bmi
D$age2 <- D$age^2
D$bmi2 <- D$bmi^2
```

We then define our lower bound as a = 0.004 and upper bound b = 0.1 for the probability of being tested, which is consistent with the lower and upper bounds of our estimated weights.

```
a <- 0.004
b <- 0.1
```

We also select our constraints and other computational options.

```
D1 <- D[dattest$sex==1,]
D0 <- D[dattest$sex==0,]

mycons1 <- list(
  list('resp', mean_response1),
  list('covmean', D1[,'age'], mean_age1),
  list('covmean', D1[,'bmi'], mean_bmi1),
  list('direc', 'positive', '+')
)

mycons0 <- list(
  list('resp', mean_response0),
  list('covmean', D0[,'age'], mean_age0),
  list('covmean', D0[,'bmi'], mean_bmi0),
  list('direc', 'positive', '+')
)

nlopts <- list("xtol_rel"=1e-8, "ftol_rel"=1e-10, "maxeval"=-1)
opts <- list("alpha2"=0.025, "grid_bound"=4, "grid_fine"=8, "tol"=1e-4, "maxeval"=3e2, "newton_step"=0.[illegible]
```

We are now ready to generate our bounds for the association between sex and testing positive. To do this we will use the `find_bound` function that is defined in the main repository (https://github.com/MRCIEU/ukbb-covid-collider).

```r
source("solve.R")
```

```r
Y1 <- dattest$positive[dattest$sex==1]
fT1 <- Y1
gT1 <- rep(1,NROW(Y1))

sens1 <- find_bound(a,b,fT1,gT1,D1,constraints=mycons1,opts,nlopts)
```

```
## [1] "Finding the nearest valid parameters from 128 starting values..."
## [1] "Found 7 valid starting parameter(s). Finding bounds..."
```

```r
sens1_upper <- sens1$beta_max + qnorm(0.9875)*sens1$se_max
sens1_lower <- sens1$beta_min - qnorm(0.9875)*sens1$se_min

Y0 <- dattest$positive[dattest$sex==0]
fT0 <- Y0
gT0 <- rep(1,NROW(Y0))

sens0 <- find_bound(a,b,fT0,gT0,D0,constraints=mycons0,opts,nlopts)
```

```
## [1] "Finding the nearest valid parameters from 128 starting values..."
## [1] "Found 4 valid starting parameter(s). Finding bounds..."
```

```r
sens0_upper <- sens0$beta_max + qnorm(0.9875)*sens0$se_max
sens0_lower <- sens0$beta_min - qnorm(0.9875)*sens0$se_min

out2 <- list(
  beta_min=sens1$beta_min-sens0$beta_max,
  beta_max=sens1$beta_max-sens0$beta_min,
  beta_lower=sens1_lower-sens0_upper,
  beta_upper=sens1_upper-sens0_lower
)

tibble(
  method="Tudball et al (2020)",
  lower_ci=round(out2$beta_lower,2),
  lower_bound=round(out2$beta_min,2),
  upper_bound=round(out2$beta_max,2),
  upper_ci=round(out2$beta_upper,2)
  ) %>% kable
```

method	lower_ci	lower_bound	upper_bound	upper_ci
Tudball et al (2020)	-0.18	-0.16	0.19	0.21

Summary

Here we have walked through a realistic example of estimating risk factors for testing positive for Covid-19 in UK Biobank. As a validation exercise, we have shown that inverse probability weighting is able to recover sample-wide associations between BMI, age and sex within the non-random sub-sample of individuals who have been tested for Covid-19. We also show that if the data is non-nested, in that a model cannot be fitted to predict participation, then summary data from a reference population can be used to generate adjusted estimates within the selected sample.

In general practice, there are a few things to further consider.

- We only used BMI, age and sex to develop weights here. In principle one would actually use as many variables as is necessary (even if they are not the exact variables being analysed) to build the model for sample selection.
- There is likely to be a change in selection pressure over time, so it is important to use weights that are relevant to the timings of the variables being recorded
- Third, in the UK Biobank the reality is slightly more complicated than has been presented here, in that the samples in the overall UK Biobank are themselves non-random (and not representative of the general population) (Munafò et al. 2018).

Supplementary references

Griffith, Gareth, Tim T Morris, Matt Tudball, Annie Herbert, Giulia Mancano, Lindsey Pike, Gemma C Sharp, et al. 2020. "Collider bias undermines our understanding of COVID-19 disease risk and severity." *medRxiv*. https://doi.org/10.1101/2020.05.04.20090506.

Groenwold, Rolf H H, Tom M Palmer, and Kate Tilling. 2020. "To adjust or not to adjust ? What to do when a 'confounder' is only measured after exposure." https://osf.io/sj7ch/.

Munafò, Marcus R, Kate Tilling, Amy E Taylor, David M Evans, and George Davey Smith. 2018. "Collider Scope: When Selection Bias Can Substantially Influence Observed Associations." *Int. J. Epidemiol.* 47 (1): 226–35.

Sayon-Orea, Carmen, Conchi Moreno-Iribas, Josu Delfrade, Manuela Sanchez-Echenique, Pilar Amiano, Eva Ardanaz, Javier Gorricho, Garbiñe Basterra, Marian Nuin, and Marcela Guevara. 2020. "Inverse-probability weighting and multiple imputation for evaluating selection bias in the estimation of childhood obesity prevalence using data from electronic health records." *BMC Medical Informatics and Decision Making* 20 (1): 9. https://doi.org/10.1186/s12911-020-1020-8.

Tudball, Matthew J, Qingyuan Zhao, Rachael A Hughes, Kate Tilling, and Jack Bowden. 2020. "An interval estimation approach to sample selection bias." https://arxiv.org/abs/1906.10159.

Zhao, Qingyuan, Dylan S Small, and Bhaswar B Bhattacharya. 2019. "Sensitivity analysis for inverse probability weighting estimators via the percentile bootstrap." https://arxiv.org/abs/1711.11286.
