# Supplementary material for: Collider bias undermines our understanding of COVID-19 disease risk and severity
Source: Nat Commun. 2020 Nov 12;11:5749. doi: 10.1038/s41467-020-19478-2 (PMC7665028; doi:10.1038/s41467-020-19478-2)
Supplement: Supplementary file 3 — Reporting Summary [file 41467_2020_19478_MOESM3_ESM.pdf]

nature research

Corresponding author(s): Gibran Hemani

Last updated by author(s): Oct 5, 2020

Reporting Summary

Nature Research wishes to improve the reproducibility of the work that we publish. This form provides structure for consistency and transparency in reporting. For further information on Nature Research policies, see our Editorial Policies and the Editorial Policy Checklist.

Statistics

For all statistical analyses, confirm that the following items are present in the figure legend, table legend, main text, or Methods section.

n/a	Confirmed	
☐	☒	The exact sample size (*n*) for each experimental group/condition, given as a discrete number and unit of measurement
☐	☒	A statement on whether measurements were taken from distinct samples or whether the same sample was measured repeatedly
☐	☒	The statistical test(s) used AND whether they are one- or two-sided<br>*Only common tests should be described solely by name; describe more complex techniques in the Methods section.*
☐	☒	A description of all covariates tested
☐	☒	A description of any assumptions or corrections, such as tests of normality and adjustment for multiple comparisons
☒	☐	A full description of the statistical parameters including central tendency (e.g. means) or other basic estimates (e.g. regression coefficient) AND variation (e.g. standard deviation) or associated estimates of uncertainty (e.g. confidence intervals)
☐	☒	For null hypothesis testing, the test statistic (e.g. *F*, *t*, *r*) with confidence intervals, effect sizes, degrees of freedom and *P* value noted<br>*Give P values as exact values whenever suitable.*
☒	☐	For Bayesian analysis, information on the choice of priors and Markov chain Monte Carlo settings
☒	☐	For hierarchical and complex designs, identification of the appropriate level for tests and full reporting of outcomes
☐	☒	Estimates of effect sizes (e.g. Cohen's *d*, Pearson's *r*), indicating how they were calculated

Our web collection on statistics for biologists contains articles on many of the points above.

Software and code

Policy information about availability of computer code

Data collection	https://github.com/MRCIEU/ukbb-covid-collider and https://github.com/explodecomputer/covid_ascertainment and https://github.com/MRCIEU/PHESANT
Data analysis	https://github.com/MRCIEU/ukbb-covid-collider and https://github.com/explodecomputer/covid_ascertainment and https://explodecomputer.github.io/AscRtain/

For manuscripts utilizing custom algorithms or software that are central to the research but not yet described in published literature, software must be made available to editors and reviewers. We strongly encourage code deposition in a community repository (e.g. GitHub). See the Nature Research guidelines for submitting code & software for further information.

Data

Policy information about availability of data

All manuscripts must include a data availability statement. This statement should provide the following information, where applicable:

- Accession codes, unique identifiers, or web links for publicly available datasets
- A list of figures that have associated raw data
- A description of any restrictions on data availability

All data analysed was provided by the UK Biobank and can be accessed via https://www.ukbiobank.ac.uk/.

Field-specific reporting

Please select the one below that is the best fit for your research. If you are not sure, read the appropriate sections before making your selection.

☒ Life sciences ☐ Behavioural & social sciences ☐ Ecological, evolutionary & environmental sciences

For a reference copy of the document with all sections, see nature.com/documents/nr-reporting-summary-flat.pdf

Life sciences study design

All studies must disclose on these points even when the disclosure is negative.

Sample size	Used all available data in UK Biobank after PHESANT processing, yielding 492,392 samples, to maximise statistical power. The rationale here is to avoid arbitrary restriction of the dataset, and to analyse the largest available dataset.
Data exclusions	Data exclusions made as described in the generation of the PHESANT pipeline, which is a preceding publication. The only samples that are excluded are those who have withdrawn consent or who don't have non-missing values for the variables analysed.
Replication	Replication was not performed. This analysis was used to establish distributions of test statistics given a particular sampling frame. There are no other datasets available to us that have similar sample sizes and with a similar sampling frame, and so replication is not possible.
Randomization	Randomization was not performed as this is an observational study design
Blinding	This is an observational study design, individual identifiers are anonymised.

Reporting for specific materials, systems and methods

We require information from authors about some types of materials, experimental systems and methods used in many studies. Here, indicate whether each material, system or method listed is relevant to your study. If you are not sure if a list item applies to your research, read the appropriate section before selecting a response.

Materials & experimental systems

n/a	Involved in the study	
☒	☐	Antibodies
☒	☐	Eukaryotic cell lines
☒	☐	Palaeontology and archaeology
☒	☐	Animals and other organisms
☐	☒	Human research participants
☒	☐	Clinical data
☒	☐	Dual use research of concern

Methods

n/a	Involved in the study	
☒	☐	ChIP-seq
☒	☐	Flow cytometry
☒	☐	MRI-based neuroimaging

Human research participants

Policy information about studies involving human research participants

Population characteristics	UK Biobank participants, described in detail here: https://www.nature.com/articles/s41586-018-0579-z
Recruitment	UK Biobank participants, described in detail here: https://www.nature.com/articles/s41586-018-0579-z
Ethics oversight	UK Biobank, described in detail here: https://www.nature.com/articles/s41586-018-0579-z

Note that full information on the approval of the study protocol must also be provided in the manuscript.
